# Plasticity of the thermal requirements of exotherms and adaptation to environmental conditions

**DOI:** 10.1002/ece3.1170

**Published:** 2014-07-15

**Authors:** Alois Honek, Zdenka Martinkova, Jan Lukas, Anthony F G Dixon

**Affiliations:** 1 Crop Research InstituteCZ 16106 Prague 6, Ruzyne, Czech Republic; 2 School of Biological Sciences, University of East AngliaNorwich, NR4 7TJ, UK

**Keywords:** Adaptation, domestication, life history, seed size, taxonomic affiliation, thermal window, thermoregulation

## Abstract

In exothermal organisms, temperature is an important determinant of the rate of ecophysiological processes, which monotonically increase between the minimum (*t*_d min_) and maximum (*t*_d max_) temperatures typical for each species. In insects, *t*_d min_ and *t*_d max_ are correlated and there is a approximately 20°C interval (thermal window *W*_T_ = *t*_d max_ − *t*_d min_) between them over which insects can develop. We assumed that other exotherms have similar thermal windows because the thermal kinetics of their physiological processes are similar. In this study, we determined the thermal requirements for germination in plants. Seeds of 125 species of Central European wild herbaceous and crop plants were germinated at nine constant temperatures between 5 and 37°C, and the time to germination of 50% of the seeds *D* and rate of germination *R* (=1/*D*) were determined for each temperature and the Lactin model used to determine *t*_d min_, *t*_d max_, and *W*_T_. The average width of the thermal windows for seeds was significantly wider (mean 24°C, 95% CI 22.7–24.2°C), varied more (between 14.5 and 37.5°C) and development occurred at lower temperatures than recorded for insects. The limiting temperatures for germination, *t*_d min_ and *t*_d max_, were not coupled, so the width of the thermal window increased with both a decrease in *t*_d min_ and/or increase in *t*_d max_. Variation in *W*_T_ was not associated with taxonomic affiliation, adult longevity, or domestication of the different species, but tends to vary with seed size. Plants are poor at regulating their temperature and cannot move to a more suitable location and as a consequence have to cope with wider ranges in temperatures than insects and possibly do this by having wider thermal windows. *Synthesis*: The study indicated specificity of *W*_T_ in different exotherm taxa and/or their development stages.

## Introduction

Temperature is an important environmental factor affecting life history traits and in particular the rate of physiological processes in all living organisms. Organic development is a vital process, which involves both tissue growth and differentiation (Dixon et al. 1982; Hopkins and Hüner 2008), and occurs over a narrow range of temperatures defined by the minimum temperature (*t*_d min_) below which development does not occur and an upper temperature that is lethal for organisms and therefore designated *t*_d let_. Most of this temperature range is made up of the “thermal window”, the range in temperature between *t*_d min_ and maximum temperature (*t*_d max_) at which development is fastest, which is usually near to the upper lethal temperature (Dixon et al. 2009). Over the range of temperatures within the thermal window, the rate of development and other vital processes increase monotonically.

Insects are typically highly mobile terrestrial exothermic animals and have a thermal window that is approximately 20°C wide (Dixon et al. 2009) regardless of whether it is located low or high on the temperature scale. This relatively narrow range of temperatures over which an insect can develop is adapted to the thermal conditions prevailing at their place of residence, geographic location or when they are active during the course of a year. The existence of a thermal window indicates that the ability of organisms to cope with variations in temperature is limited. It has been known for a long time (Belehradek 1930) that the reactions to temperature of living organisms are determined by the thermal kinetics of physiological processes, which are similar in many taxa. This expected universality of the thermal reaction of organisms is strengthened by the fact that a 20°C thermal window exists in hyperthermophile Bacteria and Archea (Madigan et al. 2000), which represent progenitors of life (Barton et al. 2007). The thermal kinetics of the physiological processes and mechanisms that determine thermal reactions might persist in descendent forms of exothermic organisms. Thus, the expectation was that the thermal reactions of other exotherms, particularly plants, would parallel those of insects (Dixon et al. 2009). However, thermal windows may also reflect adaptation to life strategies of particular taxa. Insects can mitigate the adverse effects of temperature by thermoregulation (Dixon and McKay 1970; Dixon 1972; Chown and Nicolson 2004) and in this way not only escape the adverse effects of extreme temperatures in space and time but also regulate their rate of development (Honek and Sramkova 1976). In contrast, plants and other immobile organisms are at the mercy of the thermal environment, which at certain places and times may be harsh. It was therefore decided to determine the thermal responses of plants by recording the effect of temperature on germination. Germination was selected because under constant moisture conditions, its rate is determined only by temperature, whereas the later stages of development of plants are affected by a complex of abiotic (e.g., light, moisture, temperature) and biotic (e.g., competition) factors that make measurement of thermal effects difficult.

There are many studies on the change in the rate of germination with increase in temperature, which reveal that the thermal responses of species and local populations of plants vary (Thompson 1970). The relationship between the rate of germination and temperature can be described by a linear model (Trudgill et al. 2000; Sakanoue 2010), which characterizes the germination response over the range of temperatures within the thermal window. This model provides estimates of the following thermal characteristics of germination, lower developmental threshold LDT (or basal temperature *T*_b_ and *t*_d min_), and sum of effective temperatures SET (or DD), which can be used to predict the time to germination at constant and oscillating temperatures and compare taxonomical, geographical, and ecological variation in these thermal responses (Trudgill et al. 2005). The hill-shaped relationship between rate of germination and temperature is well established and widely used for predicting the seasonal activity of plants (Grundy 2003). It is described by separate linear regressions of the ascending and descending sections of the hill-shaped function fitted to the data for the time required for 50% germination (Garcia-Huidobro et al. 1982; Hardegree et al. 1999) or by a second-order polynomial function (Cho et al. 2009). To standardize calculation of the width of thermal windows, Dixon et al. (2009) used the nonlinear models of Lactin et al. (1995) and Briere et al. (1999), of which the former gives a good fit to data on seed germination (Martinkova et al. 2006).

In this paper, we investigated the variation in thermal characteristics of germination of wild herbaceous and crops plants of the temperate zone, while keeping other determinants of seed germination, such as light and water availability, constant (Baskin and Baskin 1998). We proposed the hypothesis that the width of the thermal window is 20°C and similar to that recorded for exothermic animals. We tested whether the thermal characteristics of germination are associated with (i) the taxonomic affiliation, (ii) domestication, (iii) seed size, or (iv) life histories of plants.

## Materials and Methods

### Seed

The seeds of species of plants were selected for inclusion in this study based on the fact that percentage germination in the light at 20°C was ≥15%. This was performed as follows: Seeds of 129 herbaceous species of plants were collected in the field and stored dry as recommended by Hendry and Grime (1993) in a room at 25°C and 40% r.h. for 5–7 months (this short term after ripening might have terminated the innate dormancy of some species of seed). After this, the dry seeds were sealed in plastic bags and kept at 5°C until used in this experiment. Seeds of the 84 species that germinated after the above treatment were included in this study. Seeds of the 45 species that did not germinate were stratified for 3 months at 5°C in moist sand, dried, and again tested for germination. Seeds of eight of the species stratified that germinated were also included in this study. Thus the data set included results on the germination of 92 species of plants belonging to 19 families of wild herbaceous plants collected at localities in the western part of the Czech Republic, Slovakia (SK), Austria (AT) and France (F), located between 44°53′–50°48′N and 6°10′–19°24′E and at altitudes between 100 and 2400 m (Appendix S1). Seeds of another 33 species belonging to nine families, mostly cultivated plants, were purchased from seed companies. All these species were after ripened for an unknown period (but <1 year) and >80% germinated. In total, the seeds of 125 species of wild herbaceous and crop plants belonging to 22 families (Appendix S1) were included in this experiment. Seeds were characterized by their taxonomic affiliation (class, family), size (volume), adult longevity (annual, biennial, perennial), and domestication (wild or crop plants) (Appendix S1). The nomenclature used was that of Kubát et al. (2002), dimensions of seeds and characteristics of plants are those cited by Bojnansky and Fargasova (2007).

### Germination

Seeds were germinated in petri dishes (10 cm diameter, 2 cm deep) lined with filter paper (Whatman 1). Each petri dish contained 50–200 seeds, depending on seed size and the percentage that germinated (more small seeds were germinated at extreme temperatures at which a low germination rate was expected). The experiment started on November 1, 2008. Filter paper was soaked with 5 mL of tap water, and the petri dishes kept at one of nine constant temperatures 5, 9, 13, 17, 21, 25, 29, 33, or 37°C under a 16-h light: 8-h dark photoperiod. Moisture was controlled and if necessary, as when germinating large seeds at 29, 33, and 37°C, water was added two or three times per day. The seeds were thus always kept fully moistened, by placing them on fully saturated filter paper covering a 0.1- to 0.5-mm-deep layer of water. To keep the variation in experimental conditions at a minimum, all of the seeds were germinated simultaneously. Consequently, the number of replicates was limited to two petri dishes per species and temperature. This restriction was necessary to keep the experiment practicable. Germination was recorded daily (in the morning), and any seeds that had germinated and had protruding radicles were removed. The experiment was terminated when no more seeds germinated over a period of 10 days.

### Data analysis

For each species and temperature, the average time to germination *D* was the time to germination, which is the time that elapsed from the beginning of the experiment to the appearance of the radicle, for 50% of all the seeds that germinated. Germination rate *R* was the reciprocal of time to germination. For each species, the germination rate was plotted against temperature and the data fitted using the Lactin et al. (1995) model:

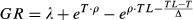


where *GR* is the mean germination rate (1/*t*_50_) at temperature *T* (°C); *ρ* is the *GR* maximum, which is reached at the optimal temperature; *TL* is the (lethal) maximum temperature; *Δ* is the temperature range over which physiological breakdown becomes the overriding influence on *GR*; and *λ* forces interception with the *x* axis, thus allowing the lower temperature threshold at which rm falls to zero to be estimated. Parameters *ρ, TL, Δ*, and *λ* were estimated using Marquardt's method and the nonlinear procedure in QC.Expert™ 2.5 (TriloByte® Ltd.) statistical software program (Kupka 2002).

**Figure 1:**
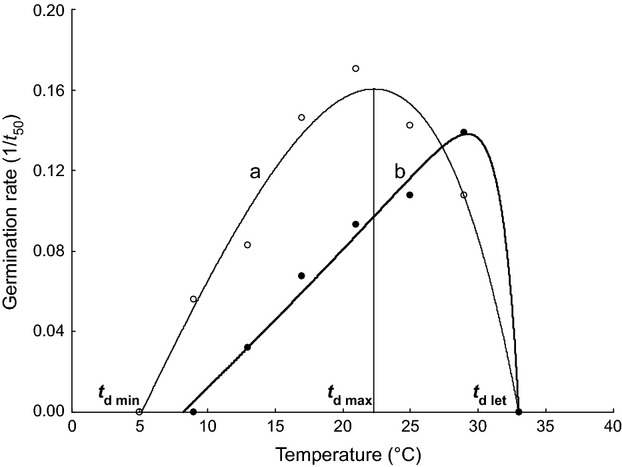
Examples of the dependency of germination rate on temperature for a – *Hieracium sabaudum* L. and b – *Hypericum maculatum* Crantz. The lines were fitted using the Lactin model, and the dots are the germination rate (based on 50% of seeds germinating) recorded at particular temperatures. The thermal characteristics *t*_d min_, *t*_d max_, and *t*_d let_ are shown for species a.

This model was used to calculate the following thermal characteristics of germination: (i) minimum temperature *t*_d min_, (ii) maximum temperature *t*_d max_, and (iii) upper “lethal” temperature *t*_d let_ (Fig. 1). This temperature, which is lethal for seeds that had started to germinate, was not lethal for dormant nongerminating seeds and certainly not for dry seeds (Martinkova and Honek 2010). Using these derived characteristics, we calculated (iv) the width of the thermal window (*W*_T_ = *t*_d max_ − *t*_d min_), (v) thermal range over which germination occurs (*t*_d let_ − *t*_d min_), and (vi) “skewness” of the germination response, which is the percentage of thermal range over which germination occurs taken up by the width of the thermal window, which is (SKEW = [(*t*_d max_ − *t*_d min_)/(*t*_d let_ − *t*_d min_)*100]).

### Linear model



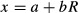


was used throughout this study to fit the data for *R* of each of the species recorded at each temperature ≤(*t*_d max_ − 1°C). Using this model, we calculated the lower temperature threshold for germination LDT = 1/*b* (Honek and Kocourek 1990), which is equivalent to *T*_b_ of Trudgill et al. (2005). Comparing the *t*_d min_ and LDT of particular species detected differences of >5°C in six species (Appendix S2). The difference was caused by the concave shape of the left ascending portion of the Lactin curve, which resulted in *t*_d min_ being incorrectly estimated. The *t*_d min_ of these species and derived characteristics calculated using *t*_d min_, *W*_T_, thermal range of germination, and skewness of the germination response, were not included in the analysis.

Within species, the mass of seeds collected from different locations may vary significantly but have little effect on their germination rate (Milberg et al. 1996; Keller and Kollmann 1999; Simons and Johnston 2000). To standardize seed size in these species, we replaced seed mass with species specific seed volume 4/3*πab*^2^ (Bojnansky and Fargasova 2007) in which *a* is the mean length and *b* the mean width of the seed. It was particularly important to standardize the size of the seed of those crop plants in which seed size may vary by a factor of 5–10 with little effect on the thermal characteristics or rate of germination (Seefeldt et al. 2002).

The arithmetic mean (±SE) values of the thermal characteristics of all the species, plant families and groups of species with similar life histories or histories of domestication studied were calculated, and the differences tested using *t*-tests (showing *p*_t_ of null hypothesis) or, when tests for normality and equal variance failed, using Mann–Whitney rank-sum test (showing *p*_MW_). Linear regression of particular thermal characteristics on other characteristics or seed size was calculated. To compensate for effect of seed size on the differences between seeds of wild herbaceous and crop plants, an analysis of covariance (ANCOVA) was used with *W*_T_ as the response variable, domestication status of species (herb vs. crop) as a factor and seed size as a covariate.

## Results

### Thermal characteristics of germination

**Figure 2:**
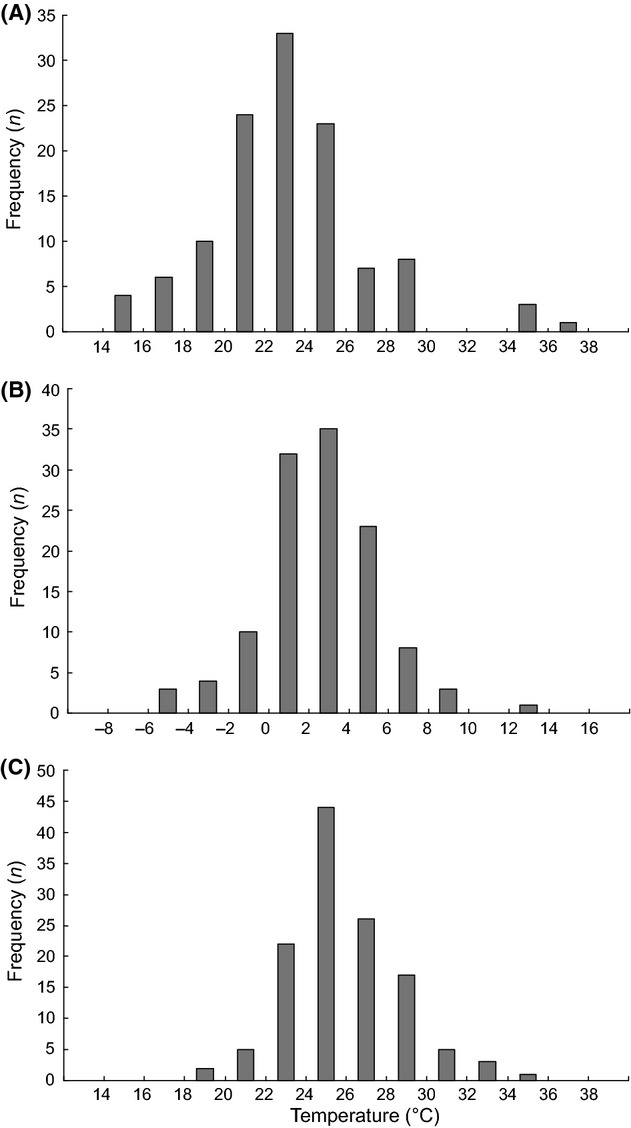
Frequency distribution of (A) the widths of the thermal windows *W*_T_ (*n* = 119), (B) minimum temperatures at which germination occurred *t*_d min_ (*n* = 119) and (C) maximum temperatures at which germination occurred *t*_d max_ (*n* = 125). In (A) and (B) six species were excluded because of uncertainty in calculating *t*_d min_ indicated by a difference of >5°C between of *t*_d min_ and LDT (see Materials and Methods). These species are indicated in Appendix S2.

**Figure 3:**
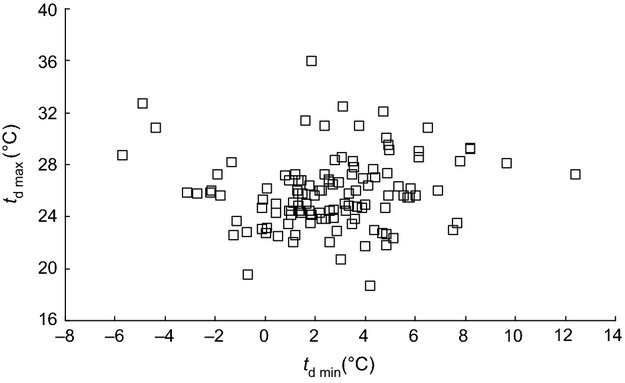
The relationship between maximum temperature at which germination occurred *t*_d max_ and the minimum temperature at which germination occurred *t*_d min_ (*P* > 0.05).

**Figure 4:**
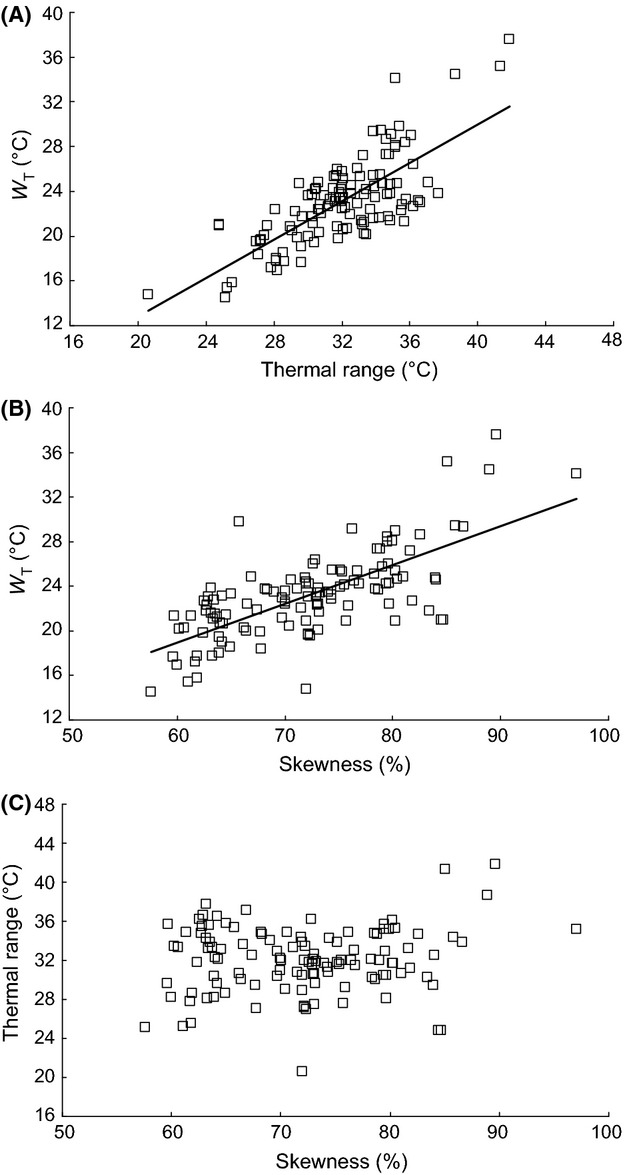
The regression relationship between (A) the width of the thermal window *W*_T_ and thermal range for germination (*a* = −4.323, *b* = 0.853, *R* = 0.747, *P* < 0.001, *n* = 119), (B) the width of the thermal window *W*_T_ and skewness of the Lactin function (*a* = −1.804, *b* = 0.346, *R* = 0.704, *P* < 0.001, *n* = 119) and (C) thermal range for germination and skewness of the Lactin function (*P* > 0.05).

**Figure 5:**
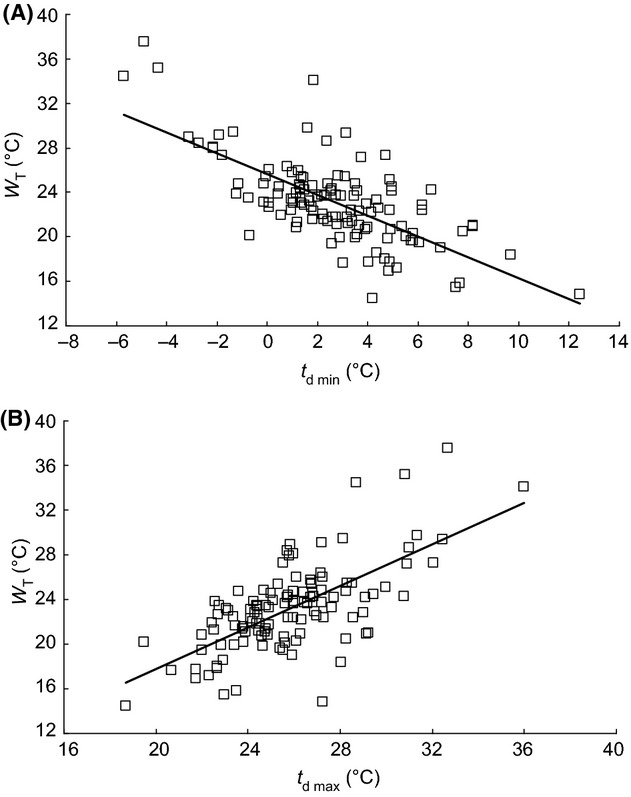
The regression relationships between (A) the width of the thermal window *W*_T_ and the minimum temperature at which germination occurred *t*_d min_ (*a* = 25.63, *b* = −0.936, *R* = 0.697, *P* < 0.001, *n* = 119) and (B) the width of the thermal window *W*_T_ and the maximum temperature at which germination occurred *t*_d max_ (*a* = −0.842, *b* = 0.931, *R* = 0.669, *P* < 0.001, *n* = 119).

The variation in size and shape of the Lactin curves (Fig. 1) indicates there is a great deal of variation in the thermal characteristics of germination (Appendix S2) of the 125 species of plants included in this study. Minimum temperature for germination, *t*_d min_, (Fig. 2B) ranged from −5.7°C in *Campanula patula* to 12.4°C in *Solanum nigrum*, with a mean of 2.6 ± 0.26°C (CI 2.1–3.1°C) and the maximum temperature, *t*_d max_, (Fig 2C) from 18.7°C in *Adenostyles alliariae* to 36.0°C in *Cerastium holosteoides,* with a mean of 25.9 ± 0.25°C (CI 25.4–26.3°C). The width of the thermal window *W*_T_ thus ranged from 14.5°C in *Adenostyles alliariae* to 37.5°C in *Carthamnus tinctorius,* with an average of 23.2 ± 0.36°C and 95% confidence intervals of 22.5–23.8°C (Fig. 2A). As the width of the thermal window *W*_T_ varied between species, *t*_d min_ and *t*_d max_ are not correlated (Fig. 3). The width of the thermal window increased as the Lactin curve became wider, that is, the thermal range for germination increased (Fig. 4A) and became more right skewed, that is, the proportion of the thermal range for germination taken up by *W*_T_ increased (Fig. 4B). Both these factors independently affect the width of the thermal window *W*_T_ (Fig. 4C). An increase in *W*_T_ was thus associated with both a decrease in the minimum temperature *t*_d min_ (Fig. 5A) and increase in the maximum temperature *t*_d max_ for germination (Fig 5B). The width of the thermal window *W*_T_ was thus flexible and extends into both low and high temperatures.

### Factors associated with the thermal responses

Thermal characteristics were not associated with the taxonomic affiliation of the different species. The 14 monocotyledenous and 111 dicotyledenous species did not differ significantly in *t*_d min_ (mean monocot 1.7 ± 0.54°C, mean dicot 2.6 ± 0.26°C, *p*_MW_ > 0.05), *t*_d max_ (26.2 ± 0.70°C, 25.8 ± 0.25°C, *p*_MW_ > 0.05) or in the width of their thermal windows *W*_T_ (24.4 ± 0.83°C, 23.2 ± 0.35°C, *p*_MW_ > 0.05). At the family level, there were no differences between the well-represented Asteraceae (32 genera and 46 species, including four crop plants) and Brassicaceae (nine genera and 11 species, including two crop plants) in *t*_d min_ (mean Asteraceae 2.8 ± 0.42°C, Brassicaceae 2.0 ± 0.71°C, *p*_t_ > 0.05), *t*_d max_ (25.2 ± 0.39°C, 24.4 ± 0.64°C, *p*_t_^ ^> 0.05), or *W*_T_ (22.4 ± 0.55°C, 22.4 ± 1.07°C, *p*_MW_ > 0.05).

Domestication appeared to be an important factor determining germination characteristics. Thermal characteristics of seeds of wild herbaceous (91 species) and crop plants (34 species) pooled across taxa significantly differed in the width of their thermal windows *W*_T_ (wild herbaceous plants 22.6 ± 0.42°C, crop plants 24.8 ± 0.61°C, *p*_MW_ < 0.001), which are associated with differences in their minimum germination temperatures *t*_d min_ (3.0 ± 0.31°C, 1.6 ± 0.45°C, *p*_MW_ < 0.05) and marginally significant differences in the “skewness” of their Lactin functions (71.4 ± 0.87%, 74.4 ± 1.15%, *p*_MW_ = 0.052). Seeds of crop plants appeared to germinate over a wider range of temperatures than those of wild herbaceous plants. However, the width of the thermal window, *W*_T,_ is also significantly positively associated with seed size when species are pooled across taxa (regression: *a* = 22.69, *b* = 0.121, *R* = 0.269, *P* < 0.005). As the seeds of wild herbaceous plants (1.9 ± 0.41 mm^3^) used in this study were significantly smaller than those of the crop plants (15.6 ± 5.72 mm^3^, *p*_MW_ < 0.001), we tested for an association with seed size in both groups. In wild herbaceous plants, there is no significant association between any of the thermal characteristics of germination and seed size. In crop plants, however, *t*_d min_ (*a* = 2.35, *b* = −0.073, *R* = 0.404, *P* < 0.05) and the width of the thermal window (*a* = 23.4, *b* = 0.133, *R* = 0.538, *P* < 0.005) are significantly associated with seed size. An ANCOVA that removed the effect of seed size revealed that the differences between wild herbaceous and crop plants in *t*_d min_ (*F*_1,116_ = 2.127, *P* > 0.05) and the width of their thermal window *W*_T_ (*F*_1,116_ = 2.802, *P* > 0.05) are not significant. The difference in the *W*_T_ of wild herbaceous and crop plants therefore is associated with differences in the size of their seed.

For the total set of species (biennials and mixed strategies excluded), the differences between annuals and perennials in *t*_d min_ (2.6 ± 0.48°C, 2.8 ± 0.44°C, *p*_t_ > 0.05), *t*_d max_ (26.7 ± 0.55°C, 25.8 ± 0.34°C, *p*_MV_ > 0.05), and width of the thermal window *W*_T_ (23.9 ± 0.81°C, 23.0 ± 0.49°C, *p*_t_ > 0.05) were not significant.

## Discussion

This paper presents the results for 125 species of herbaceous plants all germinated under the same conditions, which makes comparison easier. This was performed with the objective of comparing the width of the thermal window, *W*_T_, for insect development with that of a particular stage of plant development (germination). Although it is obvious that thermal windows exist, the question of its width is far from trivial. Some theoretical work based on thermodynamic theory predicts a universal 20°C range (Gillooly et al. 2002; Charnov and Gillooly 2003), while experimental studies on animals indicate a more plastic response in terms of the width of the thermal window in particular species (Huey and Kingsolver 1989, 1993; Frazier et al. 2006). The width of the thermal window in insects has been addressed by an analysis of published data, which indicates a mean width of 19.8°C with 95% confidence interval of 19.1–20.5°C, which is close to the theoretical prediction (Dixon et al. 2009). Nevertheless, the range in the width of the thermal window of insects is 13.3–28.6°C, which may conceal variation attributable to taxonomic and geographic constraints.

The results indicate a rather rigid determination of the width of the thermal window. The variation in the *W*_T_ of germinating seeds of herbaceous species of plants, however, may be influenced by at least two factors not addressed in this study. First, seed pretreatment may influence the width of the thermal window by affecting the intensity of dormancy. This is because termination of weak innate dormancy and germination of seeds are both controlled by temperature and may influence germination rate in opposite ways. Consequently, the width of thermal windows may be changed proportionately by the frequency of seeds that have a weak innate tendency for dormancy (Finch-Savage and Leubner-Metzger 2006). Another factor that affects “realized” width of *W*_T_ is the existence of *t*_d min_ values below 0°C. Similar negative *t*_d min_ are recorded for insects, namely larvae of stoneflies and mayflies living in cold streams (Brittain 1983). The “negative” *t*_d min_ of course do not mean that these organisms can develop and grow in ice but are active at temperatures close to 0°C. Insect larvae of cold streams even may die at temperatures of approximately +15°C. We calculated *W*_T_ using negative *t*_d mins_ and thus use the same thermal window concept as Dixon et al. (2009). A thermal window with negative values is always a virtual characteristic. Here, it should be noted that even threshold temperatures, *t*_d min,_ above 0°C cannot be experimentally approached as insects are unable to survive at temperatures 2–3°C above *t*_d min_. For insects, the inability to complete development at temperatures near to *t*_d min_ result from the loss of coordination of particular physiological and behavioral factors that are necessary for, for example, feeding or coordinated movement. Close to *t*_d min_, seeds may not germinate as well, or after such a long period of time, few are likely to survive at least under natural conditions.

The results of the current study indicate that thermal characteristics of germination differ from those of insects. The width of the thermal window for germination was 3.4°C greater than recorded for insect development (average for germination 23.2°C, *p*_MW_ < 0.001). The width is determined by *t*_d min_ (average for germination 2.6°C, insects 9.8°C, *p*_MW_ < 0.001) and *t*_d max_ (germination 25.9, insects 29.9, *p*_MW_ < 0.001), both of which are lower for germination than the development of insects.

The assumed similarity in the width of insect and plant thermal windows (Dixon et al. 2009) was thus not confirmed. The difference could be due to the taxonomic distance between these two groups of organisms, obvious differences in the physiological mechanisms regulating their thermal response, and differences in their life history strategies associated with their different levels of mobility, despite the fact that many insects and seeds are soil dwellings organisms. Moreover, the difference may be determined by the nature of the morphogenetic processes whose temperature determination is investigated. In fact, seed germination and insect development are very different physiological processes, which are hard to compare. While radicle expansion (a criterion of germination in this study) consists of cell elongation followed by cell division (Bewley and Black 1983) of a radicle of an embryo, which is already present in the seed, whereas development in insects consists of a complex sequence of morphophysiological processes. During development, insects go through a number of stages during which they develop from an egg to an adult, which involves cell division, tissue formation, and structural transformation during metamorphosis. Therefore, it is likely that these differences between seed germination and pre-adult development in insects account for the difference in the thermal responses recorded in terms of seed germination and insect development.

The question of whether the physiology of insect development and germination differ should be considered. The importance of temperature in regulating insect development and germination differs. In insects, the duration of nonfeeding stages (egg, pupa) is determined only by temperature and thus predictable from its thermal characteristics. Food quality and quantity determines the duration of development but in most cases with no effect on *t*_d min_ (Jarosik et al. 2002) and other thermal characteristics probably as well. In contrast, germination is affected by other factors. Of the intrinsic factors, seed size could be important because it is frequently associated with germination rate (Milberg et al. 1996; Simons and Johnston 2000; Zaidman et al. 2010). Extrinsic determinants are, in decreasing order of importance, water potential, changes in temperature and light intensity, and specific chemical stimuli. The shortest time to germination at a particular temperature is recorded in pure water and any decrease in water potential, by increasing osmotic pressure, increases time to germination (Bradford 2002; Easton and Kleindorfer 2009; Derbel et al. 2010). Alternating temperatures (McDonald 2002) and light intensities may increase, decrease, or have no effect on the time to germination compared to that recorded under constant temperature or lighting conditions. Consequently, thermal adaptation is only part of the strategy determining the time to germination. Results obtained by measuring the thermal characteristics of germination of species in fully hydrated conditions may not be ecologically relevant for species that live in dry habitats.

Another important difference is their ability to thermoregulate, which is important in insects but not available to seeds. Even minute insects living on the surface of the ground are capable of moving and selecting a place in which to survive the extremes of daily variation in temperature or shelter when in an immobile stage such as an egg or pupa (Honek and Sramkova 1976; Chown and Nicolson 2004). The same is true for at least some species living on the surfaces of plants (Dixon and McKay 1970; Dixon 1972; Toolson and Toolson 1991; Sanborn et al. 1992). By changing their color, position, or panting these species maintain their body temperatures within the 20°C window of “ecologically significant” temperatures. Plants need to be able to tolerate a wider range of temperatures because once seeds fall to the ground, they may be passively transported by animals or soil movements but generally cannot move elsewhere. That is, their ability to select a more suitable location is severely limited and the only choice they have is whether to germinate or not. Although the mechanism determining the time of germination can be very subtle (Baskin and Baskin 1998), once initiated, a wide tolerance of environmental conditions would be advantageous.

The differences in the thermal reactions of insects and seeds are associated with first a difference in the average *t*_d min_ which is 7°C lower in seeds than insects and second a decoupling of *t*_d min_ and *t*_d max_ in seeds, which unlike in insects, are not correlated. This is also associated with an extension in the thermal range over which germination occurs and varying skewness of their Lactin curves. There is no systematic comparison of the shapes of thermal reactions. However, for germination, the shape of the Lactin curve may vary from nearly symmetrical to highly right skewed and may differ even at the subspecific level (Qiu et al. 2010). Minor changes in the thermal response to changes in temperature during germination may result in substantial changes in width of the thermal window. The biological meaning of this fine adjustment in the width of the thermal window remains to be studied.

The limits to the variation in different taxonomic units of plants remain poorly understood, and the meaning of the variation in *W*_T_ was not revealed by this study. Testing for taxonomic diversification at the level of class (Monocotyledonae vs. Dicotyledonae) or family (Asteraceae vs. Brassicaceae) in the results of this study did not reveal any differences. The width of the thermal window was thus not constrained by taxa but correlated with ecological requirements of the species of plant. Thermal characteristics of germination are associated with seed size (Thompson et al. 1993, 2001; Hodkinson et al. 1998). Thermal requirements of species, thus, could be consistent with their ecological requirements. This was not demonstrated by this study probably because less than 5% of the species of herbaceous plants in Central Europe were included in this study. An extensive study of the British flora (Grime et al. 1981) using different methods for determining thermal characteristic revealed that the variation in thermal requirements of species is associated with their life form and habitat specialization. On the other hand, selection for traits not related to thermal adaptation, as occurs in crop breeding, does not result in changes in *t*_d min_ and probably other thermal characteristics of cultivars (Saatkamp et al. 2011). The fact that the differences in *t*_d min_ disappeared when seed size was taken into account is probably related to the fact that artificial selection for both larger seeds and a wider thermal window for germination has occurred. The mechanism of adaptation to thermal conditions needs further study.

This study indicates that the thermal requirements for development in insects and germination differ. Although the width of thermal window seems to be relatively constant in several different taxa, some plasticity in the thermal requirements for germination is advantageous because plants have to tolerate the local conditions as they cannot move elsewhere. Determining the mechanisms and adaptive significance of the variation in the width of thermal window in plants needs further study.

## Supporting Information

Appendix S1 List of the species of plants the seed of which were included in this study, their origin and characteristics.

Appendix S2 Thermal characteristics of germination – minimum temperature (*t*_d min_), maximum temperature (*t*_d max_) and lethal temperature (*t*_d let_) for germination, thermal window (*W*_T_ = *t*_d max_ − *t*_d min_), thermal range (RANG = *t*_d let_ − *t*_d min_) and “skewness” of the germination response, which is the percentage of the thermal range over which germination percentage increases (SKEW = [(*t*_d max_ − *t*_d min_)/(*t*_d let_ − *t*_d min_)*100]), which were all calculated using the Lactin function.
