# Supplementary material for: Plasticity of the thermal requirements of exotherms and adaptation to environmental conditions
Source: Ecol Evol. 2014 Jul 15;4(15):3103–12. doi: 10.1002/ece3.1170 (PMC4161183; doi:10.1002/ece3.1170)
Supplement: Appendix S1 — List of the species of plants the seed of which were included in this study, their origin and characteristics. [file ece30004-3103-sd1.doc]

Appendix 1 List of the species of plants the seed of which were included in this study, their origin and characteristics. DOM – domestication, PRE – pretreatment , LAT – geographical latitude, LON – geographical longitude, ALT – altitude of the site where collected, DAT – date of collection, ANN – annual, BIE – biennial, PER – perennial life strategy

| Species | DOM^1)^ | PRE^2)^ | Origin | LAT | LON | ALT | DAT | VOL | ANN ^5)^ | BIE ^5)^ | PER ^5)^ |
| --- | --- | --- | --- | --- | --- | --- | --- | --- | --- | --- | --- |
| **Apiaceae** |  |  |  |  |  |  |  |  |  |  |  |
| *Anethum graveolens* L. | C | A | purchased ^3)^ |  |  |  | 2008 | 5.60 | * |  |  |
| *Apium* *graveolens* L. | C | A | purchased ^3)^ |  |  |  | 2008 | 0.59 |  | * |  |
| *Daucus* *carota* L. | C | A | purchased ^3)^ |  |  |  | 2008 | 21.21 | * | * |  |
| *Foeniculum* *vulgare* Mill. | C | A | purchased ^3)^ |  |  |  | 2008 | 24.54 |  | * | * |
| *Petroselinum* *crispum* (Mill.) A.W.Hill | C | A | purchased ^3)^ |  |  |  | 2008 | 2.21 |  | * |  |
| **Asteraceae** |  |  |  |  |  |  |  |  |  |  |  |
| *Achillea* *millefolium* L. | W | A | Srbsko | 49°56‘ | 14°08‘ | 200 | 17/10/2004 | 0.36 |  |  | * |
| *Achillea* *patramica* L. | W | A | Příbram | 49°41‘ | 13°59‘ | 400 | 30/10/2004 | 1.46 |  |  | * |
| *Adenostyles* *alliariae* (Gouan) Kerner | W | A | Karlova Studánka | 50°04‘ | 17°18‘ | 1100 | 23/08/2008 | 1.91 |  |  | * |
| *Adenostyles* *leucophylla* (Willd.) Rchb. | W | S | Mtgne Ecrins (F) ^4)^ | 44°53‘ | 6°10‘ | 2400 | 01/10/2008 | 1.91 |  |  | * |
| *Arnica* *montana* L. | W | A | Knittelfeld (AT) ^4)^ | 47°13‘ | 14°50‘ | 2100 | 02/08/2007 | 3.67 |  |  | * |
| *Artemisia* *vulgaris* L. | W | A | Prague | 50°05‘ | 14°18‘ | 300 | 01/10/2004 | 0.32 |  |  | * |
| *Aster* x *salignus* Willd. | W | A | Prague | 50°05‘ | 14°18‘ | 300 | 12/10/2004 | 0.57 |  |  | * |
| *Bellis* *perennis* L. | W | A | Prague | 50°05‘ | 14°18‘ | 300 | 07/10/2004 | 0.50 |  |  | * |
| *Bidens* *pilosa* L. | W | A | purchased |  |  |  | 05/11/2006 | 0.36 | * |  |  |
| *Carduus* *acanthoides* L. | W | A | Prague | 50°05‘ | 14°18‘ | 300 | 19/10/2004 | 2.49 |  | * |  |
| *Carduus* *crispus* L. | W | A | Javory | 50°43‘ | 14°08‘ | 400 | 24/10/2004 | 3.28 |  | * |  |
| *Carthamnus* *tinctorius* L. | C | A | purchased ^3)^ |  |  |  | 2008 | 59.73 | * |  |  |
| *Centaurea* *cyanus* L. | W | A | Křeč | 49°23‘ | 14°54‘ | 500 | 15/08/2001 | 8.63 | * | * |  |
| *Centaurea* *jacea* L. | W | A | Příbram | 49°41‘ | 13°59‘ | 400 | 13/09/2006 | 2.39 |  |  | * |
| *Centaurea* *pseudophrygia* C.A.Meyer | W | A | Part. Lupča (SK) ^4)^ | 49°01‘ | 19°24‘ | 600 | 15/08/2006 | 7.56 |  |  | * |
| *Centaurea* *stoebe* L. | W | A | Přelouč | 50°02‘ | 15°34‘ | 200 | 19/11/2006 | 2.39 |  | * |  |
| *Cichorium* *intybus* L. | C | A | Prague | 50°05‘ | 14°18‘ | 300 | 31/10/2001 | 2.12 |  |  | * |
| *Cirsium* *heterophyllum* (L.) Hill | W | A | Prague | 50°05‘ | 14°18‘ | 300 | 17/08/2006 | 4.31 |  |  | * |
| *Cirsium* *oleraceum* (L.) Scop. | W | A | Javory | 50°43‘ | 14°08‘ | 400 | 24/10/2004 | 6.97 |  |  | * |
| *Cirsium* *vulgare* (Savi) Ten. | W | A | Příbram | 49°41‘ | 13°59‘ | 400 | 21/09/2003 | 2.26 |  | * |  |
| *Conyza* *canadensis* (L.) Cronquist | W | A | Prague | 50°05‘ | 14°18‘ | 300 | 02/10/2004 | 0.08 | * | * |  |
| *Crepis* *biennis* L. | W | S | Prague | 50°05’ | 14°18‘ | 300 | 05/09/2008 | 1.03 |  | * |  |
| *Echinops* *sphaerocephalus* L | W | A | Prague | 50°05‘ | 14°18‘ | 300 | 02/10/2004 | 28.65 |  |  | * |
| *Erigeron* *acris* L. | W | A | Srbsko | 49°56‘ | 14°08‘ | 200 | 17/10/2004 | 0.17 | * | * |  |
| *Galinsoga* *parviflora* Cav. | W | A | Prague | 50°05‘ | 14°18‘ | 300 | 11/10/2004 | 0.28 | * |  |  |
| *Helianthus* *annuus* L. | C | A | purchased ^3)^ |  |  |  | 2008 | 188.50 | * |  |  |
| *Hieracium* *aurantiacum* L. | W | A | Prague | 50°05‘ | 14°18‘ | 300 | 25/06/2004 | 0.20 |  |  | * |
| *Hieracium* *murorum* L. | W | A | Knittelfeld (AT) ^4)^ | 47°13‘ | 14°50‘ | 2100 | 02/08/2007 | 0.48 |  |  | * |
| *Hieracium* *pilosella* L. | W | A | Tis u Blatna | 50°05‘ | 13°20‘ | 400 | 03/07/2005 | 0.29 |  |  | * |
| *Hieracium* *sabaudum* L. | W | A | Poříčany | 50°07‘ | 14°55‘ | 200 | 03/10/2004 | 0.90 |  |  | * |
| *Hypochareis* *radicata* L. | W | A | Kralovice | 50°00‘ | 13°29‘ | 400 | 27/07/2002 | 1.19 |  |  | * |
| *Inula* *britannica* L. | W | A | Geistthal (AT) ^4)^ | 47°12‘ | 15°04‘ | 1400 | 09/08/2006 | 0.24 |  | * |  |
| *Lactuca* *sativa* L. | C | A | purchased ^3)^ |  |  |  | 2008 | 4.41 | * | * |  |
| *Lactuca* *tatarica* (L.) C.A.Meyer | W | A | Prague | 50°05‘ | 14°18‘ | 300 |  | 2.88 |  |  | * |
| *Lapsana* *communis* L. | W | A | Druzcov | 50°43‘ | 15°00‘ | 300 | 29/07/2007 | 1.06 | * |  |  |
| *Leontodon* *hispidus* L. | W | A | Židlov | 50°37‘ | 14°51‘ | 300 | 21/06/2008 | 1.80 |  |  | * |
| *Matricaria* *recutita* L. | W | A | Dírná | 49°15‘ | 14°51‘ | 400 | 19/06/2005 | 0.13 | * |  |  |
| *Matricaria* *discoidea* DC. | W | A | Dírná | 49°15‘ | 14°51‘ | 400 | 19/06/2005 | 0.11 | * |  |  |
| *Pyrethrum* *corymbosum* (L.) Scop. | W | A | Donovaly (SK) ^4)^ | 48°51‘ | 19°16‘ | 1700 | 16/08/2006 | 1.04 |  |  | * |
| *Senecio* *vulgaris* L. | W | A | Prague | 50°05‘ | 14°18‘ | 300 | 25/10/2004 | 0.36 | * |  |  |
| *Solidago* *canadensis* L. | W | A | Srbsko | 49°56‘ | 14°08‘ | 200 | 17/10/2004 | 0.05 |  |  | * |
| *Sonchus* *asper* (L.) Hill | W | A | Prague | 50°05‘ | 14°18‘ | 300 | 01/10/2004 | 3.77 | * | * |  |
| *Sonchus* *oleraceus* L | W | A | Nelahozeves | 50°16‘ | 14°19‘ | 200 | 28/10/2004 | 1.57 | * | * |  |
| *Taraxacum* *officinale* Weber ex Wiggers | W | A | Prague | 50°05‘ | 14°18‘ | 300 | 21/10/2001 | 1.94 |  |  | * |
| *Tragopogon* *pratensis* L. | W | A | Prague | 50°05‘ | 14°18‘ | 300 | 20/07/2006 | 20.03 | * |  |  |
| *Tripleurospermum* *in*odorum (L.) Schultz-Bip. | W | A | Prague | 50°05‘ | 14°18‘ | 300 | 27/08/2001 | 2.36 | * |  |  |
| **Boraginaceae** |  |  |  |  |  |  |  |  |  |  |  |
| *Myosotis* *arvensis* (L.) Hill | W | A | Kozojedy | 50°15‘ | 13°50‘ | 400 | 15/07/2001 | 0.44 | * | * |  |
| **Brassicaceae** |  |  |  |  |  |  |  |  |  |  |  |
| *Alyssum* *murale* W. et K. | W | A | Prague | 50°05‘ | 14°18‘ | 300 | 18/10/2006 | 1.00 | * |  |  |
| *Alyssum* *alyssoides* (L.) L. | W | A | Luka | 50°09‘ | 13°20‘ | 500 | 03/07/2005 | 9.65 |  |  | * |
| *Arabidopsis* *thaliana* (L.) Heynh. | W | A | Káciň | 49°39‘ | 14°07‘ | 500 | 01/11/2007 | 0.04 | * | * |  |
| *Brassica* *napus* L. | C | A | purchased ^3)^ |  |  |  | 2008 | 3.58 | * | * |  |
| *Camelina* *microcarpa* DC. | W | A | Raná | 50°25‘ | 13°47‘ | 300 | 13/09/2005 | 0.36 | * |  |  |
| *Descurainia* *sophia* (L.) Prantl | W | A | Prague | 50°05‘ | 14°18‘ | 300 | 30/07/2004 | 0.15 | * |  |  |
| *Erophila* *verna* (L.) DC | W | A | Kamenné Žehrovice | 50°08‘ | 13°55‘ | 300 | 24/04/2005 | 0.04 | * |  |  |
| *Erysimum* *crepidifolium* Rchb. | W | A | Raná | 50°25‘ | 13°47‘ | 300 | 13/09/2005 | 0.45 |  | * | * |
| *Erysimum* *hieracifolium* L. | W | A | Srbsko | 49°56‘ | 14°08‘ | 200 | 10/10/1996 | 0.54 |  | * |  |
| *Lepidium* *campestre* (L.) R. Br. | W | A | Měcholupy | 50°15‘ | 13°34‘ | 200 | 19/05/2001 | 2.77 | * | * |  |
| *Raphanus* *sativus* L. | C | A | purchased ^3)^ |  |  |  | 2008 | 9.01 | * | * |  |
| **Campanulaceae** |  |  |  |  |  |  |  |  |  |  |  |
| *Campanula* *barbata* L. | W | A | Graz (AT) ^4)^ | 47°18‘ | 15°12‘ | 1200 | 06/08/2007 | 0.19 |  |  | * |
| *Campanula* *patula* L. | W | A | Donovaly (SK) ^4)^ | 48°51‘ | 19°16‘ | 1700 | 16/08/2006 | 0.04 |  |  | * |
| *Campanula* *rotundifolia* L. | W | A | Příbram | 49°41‘ | 13°59‘ | 400 | 10/09/2006 | 0.07 |  |  | * |
| *Campanula* *trachelinum* L. | W | A | Prague | 50°05‘ | 14°18‘ | 300 | 12/09/2006 | 0.37 |  |  | * |
| **Cannabaceae** |  |  |  |  |  |  |  |  |  |  |  |
| *Cannabis* *sativa* L. | C | A | purchased ^3)^ |  |  |  | 2008 | 23.56 | * |  |  |
| **Caryophyllaceae** |  |  |  |  |  |  |  |  |  |  |  |
| *Cerastium* *glutinosum* Fries | W | A | Tušimice | 50°37‘ | 13°36‘ |  | 20/05/2001 | 0.05 | * |  |  |
| *Cerastium* *holosteoides* Fries | W | A | Prague | 50°05‘ | 14°18‘ | 300 | 04/06/2002 | 0.13 | * |  |  |
| *Dianthus* *carthusianorum* L. | W | A | Lipt. Osada (SK) ^4)^ | 48°57‘ | 19°15‘ |  | 15/08/2006 | 3.14 |  |  | * |
| *Scleranthus* *annuus* L. | W | A | Staré Město p. S. | 50°09‘ | 16°57‘ | 500 | 23/07/2006 | 0.97 | * | * |  |
| *Silene* *noctiflora* L. | W | A | Prague | 50°05‘ | 14°18‘ | 300 | 19/09/2006 | 1.38 | * |  |  |
| *Silene* *vulgaris* (Moench) Garcke | W | A | Gabčíkovo (SK) ^4)^ | 47°51‘ | 17°34‘ | 100 | 20/08/2006 | 1.41 |  |  | * |
| *Spergula* *arvensis* L. | W | A | Čelivo | 49°42‘ | 14°49‘ | 400 | 05/10/2002 | 0.45 | * |  |  |
| *Stellaria* *media* (L.) Vill. | W | A | Prague | 50°05‘ | 14°18‘ | 300 | 10/05/2001 | 0.52 | * | * |  |
| **Chenopodiaceae** |  |  |  |  |  |  |  |  |  |  |  |
| *Atriplex* *oblongifolia* W. et K. | W | S | Prague | 50°05‘ | 14°18‘ | 300 | 15/10/2006 | 1.51 | * |  |  |
| Atrip*l*ex *sagittata* Borkh. | W | S | Prague | 50°05‘ | 14°18‘ | 300 | 08/11/2006 | 2.95 | * |  |  |
| *Chenopodium* *album* L. | W | S | Prague | 50°05‘ | 14°18‘ | 300 | 15/09/2006 | 1.54 | * |  |  |
| *Chenopodium* *pumilio* R. Br. | W | A | Prague | 50°05‘ | 14°18‘ | 300 | 15/10/2006 | 0.17 | * |  |  |
| **Fabaceae** |  |  |  |  |  |  |  |  |  |  |  |
| *Lens* *culinaris* Med. | C | A | purchased ^3)^ |  |  |  | 2008 | 23.56 | * |  |  |
| *Lupinus* *polyphyllus* Lindl | C | A | purchased ^3)^ |  |  |  | 2008 | 25.44 |  |  | * |
| *Medicago* *sativa* L. | C | A | Prague | 50°05‘ | 14°18‘ | 300 | 21/10/1996 | 2.39 |  |  | * |
| *Melilotus* *officinalis* (L.) Pallas | C | A | purchased ^3)^ |  |  |  | 2008 | 1.21 |  | * |  |
| *Trifolium* *arvense* L. | W | A | Prague | 50°05‘ | 14°18‘ | 300 | 31/10/2001 | 0.28 | * | * |  |
| *Trifolium* *pratense* L. | C | A | purchased ^3)^ |  |  |  | 2008 | 1.77 |  |  | * |
| **Hypericaceae** |  |  |  |  |  |  |  |  |  |  |  |
| *Hypericum* *maculatum* Crantz | W | A | Špindlerův Mlýn | 50°45‘ | 15°38‘ | 1100 | 12/10/2006 | 0.11 |  |  | * |
| *Hypericum* *perforatum* L. | W | A | Prague | 50°05‘ | 14°18‘ | 300 | 31/10/2001 | 0.17 |  |  | * |
| **Juncaceae** |  |  |  |  |  |  |  |  |  |  |  |
| *Luzula* *luzuloides* (Lamk.) Dandy et Wilmott | W | A | Pec p. Sněžkou | 50°42‘ | 15°45‘ | 700 | 11/10/2006 | 0.30 |  |  | * |
| **Lamiaceae** |  |  |  |  |  |  |  |  |  |  |  |
| *Lavandula* *angustifolia* Mill. | C | A | Prague | 50°05‘ | 14°18‘ | 300 | 15/09/2008 | 1.80 |  |  | * |
| *Melissa* *officinalis* L. | C | A | purchased ^3)^ |  |  |  | 2008 | 0.94 |  |  | * |
| **Plantaginaceae** |  |  |  |  |  |  |  |  |  |  |  |
| *Plantago* *lanceolata* L. | W | A | Prague | 50°05‘ | 14°18‘ | 300 | 22/09/2006 | 1.73 |  |  | * |
| *Plantago* *media* L. | W | A | Prague | 50°05‘ | 14°18‘ | 300 | 12/09/2006 | 1.20 |  |  | * |
| **Poaceae** |  |  |  |  |  |  |  |  |  |  |  |
| *Alopecurus* *pratensis* L. | C | A | purchased ^3)^ |  |  |  | 2004 | 4.16 | * |  |  |
| A*p*era *spica*-*venti* (L.) P.B. | W | A | purchased ^3)^ |  |  |  | 2004 | 2.26 |  |  | * |
| *Arrhenatherum* *elatius* (L.) J. Presl at C.Presl | C | A | purchased ^3)^ |  |  |  | 2004 | 0.33 |  |  | * |
| *Avena* *nuda* L. | C | A | purchased ^3)^ |  |  |  | 2008 | 0.12 | * |  |  |
| *Bromus* *hordeaceus* L. | C | A | purchased ^3)^ |  |  |  | 2004 | 0.65 | * | * |  |
| *Holcus* *lanatus* L. | C | A | purchased ^3)^ |  |  |  | 2004 | 0.28 |  |  | * |
| *Lolium* *perenne* L. | C | A | purchased ^3)^ |  |  |  | 2004 | 0.90 |  |  | * |
| *Panicum* mil*ia*ceum L. | C | A | purchased ^3)^ |  |  |  | 2008 | 6.00 | * |  |  |
| *Phleum* *pratense* L. | C | A | purchased ^3)^ |  |  |  | 2004 | 30.69 | * | * |  |
| *Poa* *annua* L. | C | A | purchased ^3)^ |  |  |  | 2004 | 11.03 | * | * |  |
| *Poa* *pratensis* L. | C | A | purchased ^3)^ |  |  |  | 2004 | 13.74 | * |  |  |
| *Secale* *cereale* L. | C | A | purchased ^3)^ |  |  |  | 2008 | 0.59 |  |  | * |
| *Sorghum* *bicolor* (L.) Moench agg. | C | A | purchased ^3)^ |  |  |  | 2008 | 5.24 |  |  | * |
| **Polygonaceae** |  |  |  |  |  |  |  |  |  |  |  |
| *Fagopyrum* *esculentum* Moench | C | A | purchased ^3)^ |  |  |  | 2008 | 36.64 | * |  |  |
| *Polygonum* *arenastrum* Bor. | W | A | Prague | 50°05‘ | 14°18‘ | 300 | 15/10/2006 | 3.82 | * |  |  |
| *Rumex* *alpinus* L. | W | A | Špindlerův Mlýn | 50°45‘ | 15°38‘ | 700 | 11/10/2006 | 3.24 |  |  | * |
| *Rumex* *obtusifolius* L. | W | A | Prague | 50°05‘ | 14°18‘ | 300 | 19/09/2001 | 2.34 |  |  | * |
| **Portulacaceae** |  |  |  |  |  |  |  |  |  |  |  |
| *Portulaca* *oleracea* L. | W | A | Prague | 50°05‘ | 14°18‘ | 300 | 14/09/2006 | 0.30 | * |  |  |
| **Primulaceae** |  |  |  |  |  |  |  |  |  |  |  |
| *Anagallis* *arvensis* L. | W | A | Prague | 50°05‘ | 14°18‘ | 300 | 10/08/2002 | 0.57 | * | * |  |
| **Resedaceae** |  |  |  |  |  |  |  |  |  |  |  |
| *Reseda* *luteola* L. | W | S | Prague | 50°05‘ | 14°18‘ | 300 | 29/10/2007 | 0.32 |  | * |  |
| **Scrophulariacea** |  |  |  |  |  |  |  |  |  |  |  |
| *Verbascum* *phlomoides* L. | W | A | Prague | 50°05‘ | 14°18‘ | 300 | 17/10/2006 | 0.09 |  | * |  |
| *Verbascum* *phoeniceum* L. | W | A | Raná | 50°25‘ | 13°47‘ | 300 | 18/09/2006 | 0.13 |  |  | * |
| *Verbascum* *densiflorum* Bertol. | W | A | Prague | 50°05‘ | 14°18‘ | 300 | 29/10/2007 | 0.23 |  | * |  |
| *Digitalis* *purpurea* L. | W | A | Kytlice | 50°48‘ | 14°32‘ | 400 | 16/10/2002 | 0.09 |  | * | * |
| *Microrrhinum* *minus* (L.) Fourr. | W | A | Prague | 50°05‘ | 14°18‘ | 300 | 10/08/2002 | 0.08 | * |  |  |
| *Scrophularia* *nodosa* L. | W | S | Poříčany | 50°07‘ | 14°55‘ | 200 | 02/10/2004 | 0.10 |  |  | * |
| **Solanaceae** |  |  |  |  |  |  |  |  |  |  |  |
| *Hyoscyamus* *niger* L. | W | S | Prague | 50°05‘ | 14°18‘ | 300 | 17/09/2006 | 1.06 | * | * |  |
| *Nicotiana* *sylvestris* Speg. & Comes | C | A | purchased ^3)^ |  |  |  | 2008 | 0.09 | * |  |  |
| *Solanum* *lycopersicum* L. | C | A | purchased ^3)^ |  |  |  | 2008 | 16.07 | * |  |  |
| *Solanum* *nigrum* L. | W | A | Prague | 50°05‘ | 14°18‘ | 300 | 18/09/1996 | 2.81 | * |  |  |
| **Urticaceae** |  |  |  |  |  |  |  |  |  |  |  |
| *Urtica* *urens* L. | W | A | Prague | 50°05‘ | 14°18‘ | 300 | 15/09/2006 | 0.29 | * |  |  |
| **Violaceae** |  |  |  |  |  |  |  |  |  |  |  |
| *Viola* *tricolor* L. | W | A | Prague | 50°05‘ | 14°18‘ | 300 | 10/08/2002 | 0.52 | * |  |  |

1) C – crop plant, W – wild herbaceous plant

2) A – after ripened, S - stratified

3) purchased from Oseva Pro s.r.o., Prague, Czech Republic

4) country of origin: (AT) – Austria, (F) – France, (SK) – Slovakia

5) * - life strategy
