# Supplementary material for: Plasticity of the thermal requirements of exotherms and adaptation to environmental conditions
Source: Ecol Evol. 2014 Jul 15;4(15):3103–12. doi: 10.1002/ece3.1170 (PMC4161183; doi:10.1002/ece3.1170)
Supplement: Appendix S2 — Thermal characteristics of germination – minimum temperature (td min), maximum temperature (td max) and lethal temperature (td let) for germination, thermal window (WT = td max − td min), thermal range (RANG = td let − td min) and “skewness” of the germination response, which is the percentage of the thermal range over which germination percentage increases (SKEW = [(td max − td min)/(td let − td min)*100]), which were all calculated using the Lactin function. [file ece30004-3103-sd2.doc]

Appendix 2 Thermal characteristics of germination – minimum temperature (t_d min_), maximum temperature (t_d max_) and lethal temperature (t_d let_) for germination, thermal window (W_T_=t_d max_ – t_d min_), thermal range (RANG= t_d let_ - t_d min_) and "skewness" of the germination response, which is the percentage of the thermal range over which germination percentage increases (SKEW=[(t_d max_ – t_d min_)/( t_d let_ – t_d min_)*100]), which were all calculated using the Lactin function. The lower development threshold (LDT) was calculated using a linear function

| Species | t_d min_ ^1)^ | t_d max_ | t_d let_ | W_T_^1)^ | RANG^1)^ | SKEW^1)^ | LDT |
| --- | --- | --- | --- | --- | --- | --- | --- |
| **Apiaceae** |  |  |  |  |  |  |  |
| *Anethum graveolens* | 1.33 | 24.79 | 33.02 | 23.5 | 31.7 | 74.0 | 2.55 |
| *Apium graveolens* | 3.03 | 20.70 | 32.66 | 17.7 | 29.6 | 59.6 | 3.07 |
| *Daucus carota* | 0.98 | 24.40 | 33.07 | 23.4 | 32.1 | 73.0 | 2.06 |
| *Foeniculum vulgare* | 1.04 | 24.12 | 33.02 | 23.1 | 32.0 | 72.2 | 1.95 |
| *Petroselinum crispum* | 0.55 | 22.45 | 33.04 | 21.9 | 32.5 | 67.4 | -1.25 |
| **Asteraceae** |  |  |  |  |  |  |  |
| *Achillea millefolium* | 6.17 | 28.57 | 36.82 | 22.4 | 30.7 | 73.1 | 6.85 |
| *Achillea patramica* | 8.21 | 29.15 | 33.00 | 20.9 | 24.8 | 84.5 | 3.95 |
| *Adenostyles alliariae* | 4.21 | 18.68 | 29.35 | 14.5 | 25.1 | 57.6 | 3.43 |
| *Adenostyles leucophylla* | 2.76 | 23.89 | 33.07 | 21.1 | 30.3 | 69.7 | 3.21 |
| *Arnica montana* | -0.67 | 19.48 | 32.77 | 20.2 | 33.4 | 60.2 | -0.88 |
| *Artemisia vulgaris* | 3.91 | 24.61 | 36.25 | 20.7 | 32.3 | 64.0 | 2.25 |
| *Aster x salignus* | 3.51 | 28.25 | 32.98 | 24.7 | 29.5 | 83.9 | 7.40 |
| *Bellis perennis* | -3.10 | 25.85 | 32.99 | 29.0 | 36.1 | 80.2 | -2.76 |
| *Bidens pilosa* | 1.83 | 23.44 | 36.06 | 21.6 | 34.2 | 63.2 | 2.47 |
| *Carduus acanthoides* | 3.50 | 23.40 | 32.89 | 19.9 | 29.4 | 67.7 | 2.47 |
| *Carduus crispus* | 1.67 | 24.93 | 32.97 | 23.3 | 31.3 | 74.3 | 2.95 |
| *Carthamnus tinctorius* | -4.88 | 32.66 | 37.00 | 37.5 | 41.9 | 89.6 | -1.88 |
| *Centaurea cyanus* | -2.70 | 25.70 | 33.02 | 28.4 | 35.7 | 79.5 | -4.09 |
| *Centaurea jacea* | 3.67 | 24.72 | 36.90 | 21.1 | 33.2 | 63.4 | 4.26 |
| *Centaurea pseudophrygia* | 4.81 | 24.64 | 36.59 | 19.8 | 31.8 | 62.4 | 5.39 |
| *Centaurea stoebe* | 5.81 | 26.11 | 36.47 | 20.3 | 30.7 | 66.2 | 5.83 |
| *Cichorium intybus* | 4.96 | 29.11 | 36.94 | 24.1 | 32.0 | 75.5 | 5.94 |
| *Cirsium heterophyllum* | 1.79 | 26.40 | 36.66 | 24.6 | 34.9 | 70.6 | 1.70 |
| *Cirsium oleraceum* | 5.36 | 26.27 | 32.99 | 20.9 | 27.6 | 75.7 | 5.25 |
| *Cirsium vulgare* | 2.94 | 26.58 | 32.99 | 23.6 | 30.1 | 78.7 | 5.38 |
| *Conyza canadensis* | 2.56 | 26.84 | 33.03 | 24.3 | 30.5 | 79.7 | 5.32 |
| *Crepis biennis* | 0.09 | 23.13 | 36.71 | 23.0 | 36.6 | 62.9 | 1.18 |
| *Echinops sphaerocephalus* | 5.79 | 25.42 | 32.98 | 19.6 | 27.2 | 72.2 | 5.51 |
| *Erigeron acris* | 1.05 | 24.45 | 32.99 | 23.4 | 31.9 | 73.3 | 1.97 |
| *Galinsoga parviflora* | 2.27 | 23.80 | 36.15 | 21.5 | 33.9 | 63.6 | 2.15 |
| *Helianthus annuus* | -1.87* | 27.27 | 32.99 | 29.1* | 34.9* | 83.6* | 5.32 |
| *Hieracium aurantiacum* | 2.81 | 28.30 | 37.06 | 25.5 | 34.3 | 74.4 | 2.27 |
| *Hieracium murorum* | 1.20 | 22.51 | 36.88 | 21.3 | 35.7 | 59.7 | -2.04 |
| *Hieracium pilosella* | 1.34 | 25.74 | 35.23 | 24.4 | 33.9 | 72.0 | 2.57 |
| *Hieracium sabaudum* | 5.15 | 22.32 | 32.99 | 17.2 | 27.8 | 61.7 | 4.55 |
| *Hypochareis radicata* | 0.95 | 23.37 | 32.95 | 22.4 | 32.0 | 70.1 | -0.14 |
| *Inula britannica* | 7.52 | 22.95 | 32.79 | 15.4 | 25.3 | 61.1 | 7.48 |
| *Lactuca sativa* | 2.29 | 25.96 | 36.94 | 23.7 | 34.7 | 68.3 | 2.80 |
| *Lactuca tatarica* | 3.20 | 24.92 | 32.89 | 21.7 | 29.7 | 73.2 | 4.42 |
| *Lapsana communis* | 4.68 | 22.68 | 32.84 | 18.0 | 28.2 | 63.9 | 3.81 |
| *Leontodon hispidus* | 4.15 | 26.34 | 33.39 | 22.2 | 29.2 | 75.9 | 3.91 |
| *Matricaria recutita* | 2.60 | 24.38 | 37.32 | 21.8 | 34.7 | 62.7 | 2.45 |
| *Matricaria discoidea* | 6.05 | 25.55 | 32.99 | 19.5 | 26.9 | 72.4 | 5.78 |
| *Pyrethrum corymbosum* | 2.72 | 26.45 | 33.00 | 23.7 | 30.3 | 78.4 | 2.39 |
| *Senecio vulgaris* | 4.01 | 24.86 | 32.97 | 20.9 | 29.0 | 72.0 | 3.84 |
| *Solidago canadensis* | 6.16 | 28.99 | 36.88 | 22.8 | 30.7 | 74.3 | 6.04 |
| *Sonchus asper* | -2.16 | 25.79 | 33.00 | 28.0 | 35.2 | 79.5 | -2.61 |
| *Sonchus oleraceus* | 0.43 | 24.25 | 33.02 | 23.8 | 32.6 | 73.1 | 0.39 |
| *Taraxacum officinale* | -1.23 | 22.58 | 36.48 | 23.8 | 37.7 | 63.1 | -2.33 |
| *Tragopogon pratensis* | 3.65 | 25.99 | 34.25 | 22.3 | 30.6 | 73.0 | 3.20 |
| *Tripleurospermum inodorum* | 3.61 | 23.83 | 36.95 | 20.2 | 33.3 | 60.6 | 3.13 |
| **Boraginaceae** |  |  |  |  |  |  |  |
| *Myosotis arvensis* | 4.90 | 27.28 | 32.99 | 22.4 | 28.1 | 79.6 | 5.02 |
| **Brasicaceae** |  |  |  |  |  |  |  |
| *Alyssum murale* | 1.47 | 24.28 | 37.01 | 22.8 | 35.5 | 63.5 | -0.93 |
| *Alyssum alyssoides* | -0.12 | 23.04 | 36.37 | 23.2 | 36.5 | 64.2 | 0.05 |
| *Arabidopsis thaliana* | -1.12 | 23.61 | 33.26 | 24.7 | 34.4 | 71.9 | -1.48 |
| *Brassica napus* | 1.38 | 24.44 | 33.00 | 23.1 | 31.6 | 72.9 | 2.11 |
| *Camelina microcarpa* | -1.32 | 28.13 | 33.00 | 29.5 | 34.3 | 85.8 | 0.14 |
| *Descurainia sophia* | 2.58 | 22.00 | 32.96 | 19.4 | 30.4 | 63.9 | 2.01 |
| *Erophila verna* | -4.79* | 24.45 | 33.04 | 29.2* | 37.8* | 77.3* | 0.70 |
| *Erysimum crepidifolium* | 3.94 | 26.89 | 36.85 | 22.9 | 32.9 | 69.7 | 3.25 |
| *Erysimum hieracifolium* | 4.36 | 22.92 | 32.93 | 18.6 | 28.6 | 65.0 | 4.11 |
| *Lepidium campestre* | 4.01 | 21.72 | 32.64 | 17.7 | 28.6 | 61.9 | 3.25 |
| *Raphanus sativus* | 4.42 | 26.98 | 36.64 | 22.6 | 32.2 | 70.0 | 4.94 |
| **Campanulaceae** |  |  |  |  |  |  |  |
| *Campanula barbata* | 1.49 | 25.72 | 33.01 | 24.2 | 31.5 | 76.9 | 0.64 |
| *Campanula patula* | -5.70 | 28.72 | 33.00 | 34.4 | 38.7 | 88.9 | -5.19 |
| *Campanula rotundifolia* | 0.80 | 27.16 | 37.00 | 26.4 | 36.2 | 72.8 | 0.24 |
| *Campanula trachelinum* | -0.06 | 25.31 | 33.01 | 25.4 | 33.1 | 76.7 | -1.27 |
| **Cannabaceae** |  |  |  |  |  |  |  |
| *Cannabis sativa* | -4.16* | 29.87 | 37.03 | 34.0* | 41.2* | 82.6* | 3.95 |
| **Caryophyllaceae** |  |  |  |  |  |  |  |
| *Cerastium glutinosum* | 3.13 | 32.46 | 37.00 | 29.3 | 33.9 | 86.6 | 3.79 |
| *Cerastium holosteoides* | 1.85 | 35.96 | 37.00 | 34.1 | 35.1 | 97.1 | 2.73 |
| *Dianthus carthusianorum* | 3.38 | 24.78 | 36.54 | 21.4 | 33.2 | 64.5 | 2.68 |
| *Scleranthus annuus* | 2.41 | 23.78 | 37.25 | 21.4 | 34.8 | 61.3 | 1.88 |
| *Silene noctiflora* | 5.72 | 25.40 | 32.98 | 19.7 | 27.3 | 72.2 | 5.37 |
| *Silene vulgaris* | 0.06 | 22.70 | 36.27 | 22.6 | 36.2 | 62.5 | 2.95 |
| *Spergula arvensis* | 3.24 | 24.45 | 36.55 | 21.2 | 33.3 | 63.7 | 0.33 |
| *Stellaria media* | 4.85 | 21.75 | 33.02 | 16.9 | 28.2 | 60.0 | 3.35 |
| **Chenopodiaceae** |  |  |  |  |  |  |  |
| *Atriplex oblongifolia* | 1.85 | 24.14 | 37.35 | 22.3 | 35.5 | 62.8 | 3.22 |
| *Atriplex sagittata* | 4.35 | 27.64 | 40.16 | 23.3 | 35.8 | 65.0 | 4.18 |
| *Chenopodium album* | 2.54 | 26.71 | 32.99 | 24.2 | 30.5 | 79.4 | 2.89 |
| *Chenopodium pumilio* | 2.37 | 30.99 | 37.03 | 28.6 | 34.7 | 82.6 | 6.89 |
| **Fabaceae** |  |  |  |  |  |  |  |
| *Lens culinaris* | -0.73 | 22.75 | 33.27 | 23.5 | 34.0 | 69.0 | -2.10 |
| *Lupinus polyphyllus* | -1.77 | 25.55 | 33.01 | 27.3 | 34.8 | 78.5 | -2.06 |
| *Medicago sativa* | -2.13 | 25.96 | 33.00 | 28.1 | 35.1 | 79.9 | -1.66 |
| *Melilotus officinalis* | -0.12 | 24.69 | 36.98 | 24.8 | 37.1 | 66.9 | -1.21 |
| *Trifolium arvense* | 3.35 | 25.75 | 37.01 | 22.4 | 33.7 | 66.5 | 2.94 |
| *Trifolium pratense* | 2.19 | 25.98 | 37.06 | 23.8 | 34.9 | 68.2 | 0.77 |
| **Hypericaceae** |  |  |  |  |  |  |  |
| *Hypericum maculatum* | 8.21 | 29.22 | 33.00 | 21.0 | 24.8 | 84.7 | 8.15 |
| *Hypericum perforatum* | 4.95 | 29.44 | 36.98 | 24.5 | 32.0 | 76.5 | 4.61 |
| **Juncaceae** |  |  |  |  |  |  |  |
| *Luzula luzuloides* | 4.88 | 22.66 | 33.00 | 17.8 | 28.1 | 63.2 | 4.01 |
| **Lamiaceae** |  |  |  |  |  |  |  |
| *Lavandula angustifolia* | 3.57 | 27.74 | 37.00 | 24.2 | 33.4 | 72.3 | 4.24 |
| *Melissa officinalis* | 7.80 | 28.26 | 36.86 | 20.5 | 29.1 | 70.4 | 7.71 |
| **Plantaginaceae** |  |  |  |  |  |  |  |
| *Plantago lanceolata* | 0.80 | 27.16 | 37.00 | 26.4 | 36.2 | 72.8 | -0.06 |
| *Plantago media* | 7.68 | 23.49 | 33.24 | 15.8 | 25.6 | 61.9 | 6.22 |
| **Poaceae** |  |  |  |  |  |  |  |
| *Alopecurus pratensis* | 0.46 | 24.99 | 33.02 | 24.5 | 32.6 | 84.1 | 0.13 |
| *Apera spica-venti* | 1.45 | 26.77 | 33.00 | 25.3 | 31.6 | 75.3 | 0.13 |
| *Arrhenatherum elatius* | -1.91 | 27.21 | 33.00 | 29.1 | 34.9 | 76.2 | 1.72 |
| *Avena nuda* | 1.15 | 22.00 | 32.87 | 20.9 | 31.7 | 80.3 | 1.44 |
| *Bromus hordeaceus* | 1.78 | 24.43 | 32.96 | 22.6 | 31.2 | 81.8 | 1.63 |
| *Holcus lanatus* | 0.99 | 26.74 | 32.99 | 25.8 | 32.0 | 79.0 | -0.59 |
| *Lolium perenne* | 2.74 | 24.53 | 33.01 | 21.8 | 30.3 | 83.4 | -2.00 |
| *Panicum miliaceum* | 1.61 | 31.37 | 37.00 | 29.8 | 35.4 | 65.7 | 0.04 |
| *Phleum pratense* | 2.01 | 25.62 | 32.99 | 23.6 | 31.0 | 70.0 | 1.83 |
| *Poa annua* | 1.24 | 27.23 | 33.00 | 26.0 | 31.8 | 72.6 | 1.05 |
| *Poa pratensis* | 0.46 | 24.99 | 33.02 | 24.5 | 32.6 | 84.1 | 0.13 |
| *Secale cereale* | 1.30 | 25.98 | 36.56 | 24.7 | 35.3 | 80.5 | 1.41 |
| *Sorghum bicolor* | 6.53 | 30.77 | 37.00 | 24.2 | 30.5 | 72.0 | 2.92 |
| **Polygonaceae** |  |  |  |  |  |  |  |
| *Fagopyrum esculentum* | 3.09 | 28.53 | 36.93 | 25.4 | 33.8 | 75.2 | 4.35 |
| *Polygonum arenastrum* | -4.46* | 29.40 | 36.99 | 33.9* | 41.4* | 81.7* | 3.48 |
| *Rumex alpinus* | -8.30* | 26.19 | 33.04 | 34.5* | 41.3* | 76.8* | 2.21 |
| *Rumex obtusifolius* | 5.54 | 25.61 | 32.99 | 20.1 | 27.4 | 73.1 | 5.22 |
| **Portulacaceae** |  |  |  |  |  |  |  |
| *Portulaca oleracea* | 4.73 | 32.04 | 39.36 | 27.3 | 34.6 | 78.9 | 6.39 |
| **Primulaceae** |  |  |  |  |  |  |  |
| *Anagallis arvensis* | 1.36 | 26.78 | 33.02 | 25.4 | 31.7 | 80.3 | 5.05 |
| **Resedaceae** |  |  |  |  |  |  |  |
| *Reseda luteola* | 2.20 | 24.23 | 32.98 | 22.0 | 30.8 | 71.6 | 3.33 |
| **Scrophulariacea** |  |  |  |  |  |  |  |
| *Verbascum phlomoides* | 4.87 | 30.00 | 36.96 | 25.1 | 32.1 | 78.3 | 6.92 |
| *Verbascum phoeniceum* | 6.91 | 25.94 | 36.55 | 19.0 | 29.6 | 64.2 | 5.78 |
| *Verbascum densiflorum* | 9.68 | 28.05 | 36.77 | 18.4 | 27.1 | 67.8 | 9.36 |
| *Digitalis purpurea* | 4.93 | 25.56 | 37.00 | 20.6 | 32.1 | 64.3 | 3.70 |
| *Microrrhinum minus* | 2.40 | 27.21 | 33.02 | 24.8 | 30.6 | 81.0 | 5.78 |
| *Scrophularia nodosa* | 3.47 | 27.18 | 36.79 | 23.7 | 33.3 | 71.2 | 3.90 |
| **Solanaceae** |  |  |  |  |  |  |  |
| *Hyoscyamus niger* | -4.34 | 30.82 | 37.00 | 35.2 | 41.3 | 85.1 | -3.54 |
| *Nicotiana sylvestris* | -6.23* | 28.29 | 33.01 | 34.5* | 39.2* | 83.1* | 6.68 |
| *Solanum lycopersicum* | 3.76 | 30.93 | 37.03 | 27.2 | 33.3 | 81.7 | 7.04 |
| *Solanum nigrum* | 12.42 | 27.23 | 33.00 | 14.8 | 20.6 | 72.0 | 12.78 |
| **Urticaceae** |  |  |  |  |  |  |  |
| *Urtica urens* | 2.89 | 22.84 | 32.92 | 19.9 | 30.0 | 66.4 | 2.51 |
| **Violaceae** |  |  |  |  |  |  |  |
| *Viola tricolor* | 1.15 | 25.08 | 32.98 | 23.9 | 31.8 | 75.2 | 0.63 |

1) * not included in the analysis
